# Lameness in dairy heifers; impacts of hoof lesions present around first calving on future lameness, milk yield and culling risk

**DOI:** 10.1016/j.prevetmed.2016.09.006

**Published:** 2016-10-01

**Authors:** L.V. Randall, M.J. Green, M.G.G. Chagunda, C. Mason, L.E. Green, J.N. Huxley

**Affiliations:** a University of Nottingham, School of Veterinary Medicine and Science, Sutton Bonington Campus, Sutton Bonington, Leicestershire, LE12 5RD, United Kingdom; b Scotland’s Rural College (SRUC), Kings Buildings, West Mains Road, Edinburgh, EH9 3JG, United Kingdom; c School of Life Sciences, University of Warwick, Coventry, CV4 7AL, England, United Kingdom

**Keywords:** Dairy heifer, Hoof lesion, Lameness, Milk yield, Culling

## Abstract

The importance of lameness in primiparous dairy heifers is increasingly recognised. Although it is accepted that clinical lameness in any lactation increases the risk of future lameness, the impact of foot lesions during the first lactation on long-term lameness risk is less clear. This retrospective cohort study aimed to investigate the impacts of foot lesions occurring around the time of first calving in heifers on future lameness risk, daily milk yield and survival within a dairy herd. Records were obtained for 158 heifers from one UK dairy herd. Heifers were examined in 2 month blocks from 2 months pre-calving through to 4 months post-calving. Sole lesions and white line lesions were scored on a zero to 10 scale and digital dermatitis on a zero to 3 scale. Outcomes investigated were; lameness risk based on weekly locomotion scores, average daily milk yield and culling risk. Mixed effect models were used to investigate associations between maximum lesion scores and outcomes. Lesion scores in the highest score categories for claw horn lesions (sole lesions and white line lesions) in the 2 to 4 month post-calving period were associated with an increased risk of future lameness; heifers with white line lesion scores ≥3 compared with scores zero to 1 and heifers with sole lesion scores ≥4 compared with score 2, at this time point, had a predicted increased risk of future lameness of 1.6 and 2.6 respectively. Sole lesions ≥4 were also associated with a reduction in average daily milk yield of 2.68 kg. Managing heifers to reduce claw horn lesions during this time period post-calving may provide health, welfare and production benefits for the long-term future of those animals. A novel finding from the study was that mild lesion scores compared with scores zero to 1, were associated with a reduced risk of future lameness for white line lesions and sole lesions occurring in the pre-calving or 2 to 4 months post-calving periods respectively. Mild sole lesions in the pre-calving period were also associated with a reduced risk of premature culling. One hypothesis for this result is that a mild insult may result in adaptive changes to the foot leading to greater biomechanical resilience and so increased longevity.

## Highlights

• More severe claw horn lesions were associated with increased risk of future lameness.
• More severe sole lesions were associated with reduction in daily yield of 2.68 kg.
• Managing heifers to reduce severe lesions is likely to have positive herd impacts.
• Mild claw horn lesions associated with reduced risk of future lameness and culling.
• Mild insult may lead to adaptive changes in the hoof which are beneficial.

## 1 Introduction

Lameness is one of the most significant diseases currently impacting on dairy cow health, welfare and productivity (Huxley, 2013). Since a first occurrence of lameness increases the future risk of lameness (Hirst et al., 2002, Green et al., 2014, Randall et al., 2015), lameness in dairy heifers has the potential to have a severe impact on their overall lifetime performance within the herd. The significance of this is most pronounced when considering the high prevalence of lesions in heifers (Manske et al., 2002, Capion et al., 2009, Maxwell et al., 2015). Capion et al. (2009) found the prevalence of moderate to severe sole haemorrhage and white line lesions in 147 Danish Holstein heifers was 55% and 72% at 1–100 days in milk (DIM) respectively and the prevalence of digital dermatitis (DD) peaked at 39% at 0–100 DIM. Similarly, Maxwell et al. (2015) reported that 95% of a cohort of 139 Holstein dairy heifers being trimmed at between 50 and 80 days post-partum had some pathology on at least one claw. Lameness in the first lactation has been associated with a doubling of the hazard for lameness in the second lactation (Hirst et al., 2002). Consequently, Bell et al. (2009) suggested that a critical control point for lameness in dairy cattle should aim to prevent claw horn lesions and digital dermatitis in heifers. The transition period, around the time of calving, has been identified as an important risk period, with increased stress related to physiological changes, social factors and changes in housing that impact on the risk of lameness occurring in heifers (Tarlton et al., 2002, Bergsten et al., 2015). Webster (2002) reported that heifers housed in straw yards for eight weeks after calving before being moved to cubicle housing resulted in less severe sole haemorrhages compared to heifers introduced to cubicle housing four weeks before calving. This finding demonstrated that housing practices around the time of calving affect the development of foot lesions in dairy heifers. The impact of lesions in heifers on long-term lameness is not yet known and could have major implications for the future health and welfare of the dairy herd.

Lameness in dairy cows has also been demonstrated to be associated with significant impacts on performance, such as reduced milk yield and increased culling risk (Booth et al., 2004, Amory et al., 2008). For other diseases, such as mastitis, it has been shown that disease occurring in heifers affects lifetime performance, for example an increase in somatic cell count in heifers in early lactation negatively impacts on lifetime milk yield (Archer et al., 2013). This relationship may also be true for lameness, but has not yet been fully explored.

This study aimed to investigate the long-term impacts of hoof lesions that occur around the time of first calving in heifers, on lameness, daily milk yield and culling risk. A retrospective cohort study using mixed effect logistic regression and linear regression models was conducted to test the null hypothesis that hoof lesions occurring around the time of first calving in heifers have no impact on future lameness risk, average daily milk yield and culling risk in one UK dairy herd.

## 2 Materials and methods

### 2.1 Study herd

Records for 158 Holstein Friesian heifers that calved for the first time between August 2003 and March 2006 were obtained from the Scotland’s Rural College (SRUC) Dairy Research and Innovation Centre in Dumfries, Scotland. Lifetime data for these animals were collected from September 2003 to August 2011. The SRUC centre has two pedigree research herds which are based at the same site; the ‘Langhill’ systems herd and ‘Acrehead’ herd. Cows remained within the Langhill herd for typically 3 lactations, after which they were moved to Acrehead, however if no replacement heifers were due to calve within 2 months, the cow remained at Langhill for one or more additional lactations (Roberts and March, 2013).

The Langhill herd was managed on a long-term 2 × 2 factorial genetic and feed management system that comprised two contrasting dairy management systems; low forage, continuously housed (LF) and high-forage, grazed (HF) groups. Cows belonging to one of two genetic lines, Control (C) and Select (S), were divided equally between the management systems (Pryce et al., 1999). These management systems are described in further detail below. The Acrehead herd was managed as a separate research and experimental herd with no long-running feed or management groups.

#### 2.1.1 Young-stock management prior to first calving

At the Langhill site, heifers calved all year round. Young-stock were reared in stable groups of approximately 25 animals from weaning to the start of their first transition period at approximately eight weeks before calving. As calves, they remained with the dam until at least 24 h of age, and were fed 2 l of colostrum by stomach tube. Following removal from the dam, calves were housed individually indoors in straw-bedded pens and received 2 l of pooled colostrum twice daily for up to 7 days, followed by 6 l per day of calf milk replacer. After ten days, calves were housed in group pens with deep straw bedding; the UK minimum recommended space allowance (Defra, 2003) was exceeded at all times. Fresh water was available from drinking bowls fitted to the wall of the building and calf milk replacer was fed via automatic feeders. Calves were weaned at approximately 50 to 60 days and managed as one group of dairy replacement young-stock; they were reared indoors until their second summer. Heifers were grazed during their second summer and then fed a young-stock ration when housed during winter. Table 1 presents a summary of the typical formulation for the young-stock ration. Housing was straw bedded pens until 12 to 15 months of age, at which time all heifers were moved to cubicle housing with mattress and sawdust until the transition period. Passageways were grooved concrete. Target age at first calving was 24 months; first service was scheduled at approximately 350 kg of BW and 15 months of age. All inseminations were artificial. No routine foot trimming was performed prior to first calving. Footbathing was carried out monthly for young-stock using 5% copper sulphate solution. Live weight was recorded monthly using walk-in weigh scales. Prior to the start of the transition period before first calving, heifers were separated according to the feeding system to which they had been allocated (described below) and were fully housed in straw bedded pens until calving. The same management protocols were applied by the same stock persons and technicians throughout the study period.

#### 2.1.2 Lesion scoring around first calving

During the period 1st September 2003 to 31st January 2006, all four feet of heifers were lifted and lesions were recorded on standardised hoof maps (Greenough and Vermunt, 1991). Examinations were carried out by the same veterinary surgeon at regular intervals, approximately two months apart, with an average of 37 heifers being examined each time over the three year period 2003 to 2006. Lesions were severity scored on a 1 to 10 scale for sole or white line lesions (1 to 5 for haemorrhage and 6 to 10 for sole ulcers or white line separation) and a 1 to 3 scale for digital dermatitis (1 for mild or 3 for severe) as described by Offer et al. (2000) and Leach et al. (2005) (Table 2).

#### 2.1.3 Management subsequent to first calving

As heifers calved they were introduced into the Langhill milking herd, remaining within the feeding system to which they had been allocated prior to calving. The detailed diet and management systems for the herd have been described by Chagunda et al. (2009). Briefly, low forage (LF) cows were housed continuously throughout the year whilst high forage (HF) cows were housed during winter months (typically November to March) and grazed during summer months provided sufficient herbage was available. When housed, cows were fed a complete diet that was between 45% and 50% forage in the dry matter (DM) for those in the continuously housed, low forage group, and 70% to 75% forage in the DM for those in the high forage group. Concentrates were included at approximately 3000 kg and 1200 kg per cow per year respectively for the low forage and high forage diets, with target yields being 13,000 kg and 7,500 kg per cow per year respectively. The herd was all-year round calving and milked three times daily. Housing was the same for cows in both the low and high forage groups; cubicles with mattresses (mats prior to 2004) and sawdust bedding. Passageways were grooved concrete and were automatically scraped every 2 h. Footbathing was carried out regularly using 5% copper sulphate solution; once weekly at 3 consecutive milking’s for lactating cows and once weekly for dry cows. A professional foot trimmer attended bi-annually to trim all four feet of the whole herd. Cows were moved to Acrehead, typically after 3 lactations, according to Langhill research herd protocol requirements. Housing and general management was the same for cows in the Acrehead herd as it was for Langhill. Cows were milked three times daily and fed a grass-silage based total mixed ration, formulated to provide adequate nutrients for maintenance and milk production. All cows were housed in cubicle housing during winter months and had the potential to graze for varying period throughout summer months (Rioja-Lang et al., 2009).

#### 2.1.4 Data collection during lifetime lactation

##### 2.1.4.1 Langhill

Locomotion scores and body condition scores were recorded weekly by experienced, trained assessors and following standard protocols. In order to reduce the impact of operator bias, assessors alternated weekly and underwent regular training with the same veterinarian during the study period. A 1 to 5 scoring scale (LS 1 to 5) was used to measure locomotion (according to Manson and Leaver (1988)). Cows recorded LS 4 or 5 on a single occasion or LS 3 on two successive occasions were examined and treated by a veterinarian; weekly prior to 2006 and every two weeks thereafter. Cows observed lame between weekly scoring were treated within 24 h by trained staff. A 0 to 5 categorical scale with increments of 0.25 was used to body condition score cows (Mulvany, 1977). Body weights were recorded after milking three times daily using an automatic weighing system. Health, production and management data were recorded in a database, including culling dates.

##### 2.1.4.2 Acrehead

Locomotion scores, body condition scores, milk yield and body weight were not systematically recorded. Culling dates were recorded within the main database.

Cows spent on average 3.9 years (3.5 lactations) within the Langhill herd, and is referred to throughout this paper as ‘herd lifetime’.

### 2.2 Statistical methods

Data recorded from heifers calving during the period August, 1, 2003 to March, 31, 2006 were obtained; lesion data recorded during the period September, 1, 2003 to January, 31, 2006 and lifetime health and production data recorded as these animals were followed through from September, 1, 2003 to August, 31, 2011. Microsoft Excel 2010 (Microsoft Corp.) was used for data handling and manipulation including identification and removal of unusual or anomalous data and constructing categorical variables. Where possible, missing observations were included as a categorical variable and fitted within each of the models to minimise the loss of data (results not reported). Three examination periods were assigned to the data according to when heifers had lesions scored in relation to calving; 0 to 2 months pre-calving, 0 to 2 months and 2 to 4 months post-calving.

Maximum, sum and mean of the scores for sole lesions, white line lesions and digital dermatitis recorded on the hind feet were calculated for each heifer for each of the three assessment points (0 to 2 months pre-calving, 0 to 2 and 2 to 4 months post-calving). Lesion scores were added to the lifetime data records for the study population using the data set previously described by Randall et al. (2015). Outcome variables of interest were; lameness (based on locomotion score) categorised as ‘not lame’ (LS 1 or LS 2), or ‘lame’ (LS 3, LS 4 or LS 5), average daily milk yield (kg) as a continuous variable and culling as a binary variable (0 or 1 for not culled or culled respectively). Lesion score categories with a similar effect on the outcome variable were grouped together to ensure the minimum number of cows within a category was 10. Average daily milk yield was calculated for the time from first calving through to removal from the Langhill herd. Kendall’s correlation coefficient (Kendall, 1955) was used to determine the correlation between sole lesions and white line lesions during different time periods.

All models were constructed in MLWin 2.28 (Rabash et al., 2009). Multilevel models were used to explore the relationship between lesion scores and the outcomes of repeated locomotion scores and survival to culling. Data were structured at the cow week level. Initial assessment of model parameters was carried out using the iterative generalised least square procedure for parameter estimation (Goldstein, 2003) with forward selection of explanatory variables. Biologically plausible interactions were investigated. Final parameter estimates for each model were made using Markov Chain Monte Carlo (MCMC) to reduce the potential for biased estimates (Rabash et al., 2009), using procedures described by Green et al. (2004). Briefly, explanatory variables remained within the model if the 95% credible interval of the odds ratio did not include 1 and as such were considered ‘significant’. A minimum burn-in of 5000 iterations was used, during which model convergence occurred. Final parameter estimates were based on a maximum of a further 50,000 iterations. Chain mixing and stability were examined visually. To explore the relationship between lesion scores and average daily milk yield, a linear regression model was used, with data structured at the cow level (Dohoo et al., 2003). Model parameters were estimated using the iterative generalised least square procedure (Goldstein, 2003) and explanatory variables remained within the model if P ≤ 0.05. A forward selection procedure was used for model building. Methods for assessing model fit and posterior predictions are described in detail below.

#### 2.2.1 Model 1: impacts of lesions present around first calving on lameness

The data were analysed as a frailty model using a mixed effect binomial logistic regression framework (Goldstein, 2003), where each cow could have repeated lameness events over time. Cow was included as a random effect and time since last lameness event as a fixed effect. This model equates to a multilevel survival model with random effects (Goldstein, 1995). The model took the form;

Lame_ij_ ∼ Bernoulli (probability = π_ij_)

Logit(π_ij_) = α + β_1_wk_ij_ + β_2_X_ij_ + β_3_X_j_ + u_j_

[u_j_] ∼ N(0, σ^2^_v_)

Where subscripts *i* and *j* denote the *i*th observation of the *j*th cow respectively. π_ij_ = probability of a lame outcome for the *i*th observation of the *j*th cow. α = intercept value, wk_ij_ = categorical variable to represent week of the study for the *i*th observation of the *j*th cow, β_1_ = vector of coefficients for wk_ij_, X_ij_ = vector of covariates associated with each observation, β_2_ = coefficients for covariates X_ij,_ X_j_ = vector of covariates associated with each cow, β_3_ = coefficients for covariates X_j_. u_j_ = random effect to account for residual variation between cows (assumed to be normally distributed with mean = 0 and variance = σ^2^_v_).

Lesion scores were included as a categorical explanatory variable. Other potentially confounding explanatory variables tested included; categories for parity (1 to 4 + ), previous LS 3, 4 or 5 (yes or no in two month intervals; 0 to 2 months previously, 2 to 4 months previously and >4 months previously), age at first calving (<24months, 24 to 27 months, 28 to 30 months, 31 to 33 months and greater than 33 months), feed-genetic group (low-forage control: LF-C, low-forage select: LF-S, high forage control: HF-C, high forage select: HF-S, dry-control: D-C, dry-select: D-S, other-control: O-C, other-select: O-S, where other represents all management groups outside of LF, HF and Dry). Locomotion score assessor was included as an explanatory variable to control for possible inter-observer variability (Locomotion score recorder; 1 to 4). Weeks in milk (WIM) was categorised in five 8-week intervals from 0 to 40 weeks and a separate category for >40 weeks. Week of the study was included as a categorical variable to account for background changes in risk over time. Within the data set there were a small number of cows with a high number of weeks recorded ‘lame’, which would influence model parameters. Therefore a term was included for cows with greater than 40 lame weeks, with the threshold value being selected based on examination of the frequency distribution of the number of lame weeks per cow.

Posterior predictions were used to assess model fit by visual comparison to the observed data (Gelman et al., 1996) and the Hosmer-Lemeshow test (Hosmer and Lemeshow, 1989) was used as a statistical test for goodness of fit for mixed effect models by comparing deciles of fitted risk values to the matched observed risk. Posterior predictions were also used to calculate relative risks for each of the lesion categories.

#### 2.2.2 Model 2: impacts of lesions present around first calving on milk yield

A linear regression model was used to analyse the data with animal average daily milk yield for time spent at Langhill, as the outcome. The model took the form;

Yield_i_ ∼ N(*XB*, Ω)

Yield_i_ = α + β_1_X_i_ + e_i_

[e_i_] ∼ N(0, Ω_e_)

Where Yield_i_ is the average daily yield for the *i*th cow. α = intercept value, β_1_ = vector of covariates associated with each cow and e_i_ represents the residual error (assumed to be normally distributed, with mean = 0 and variance = Ω_e)._

Lesion scores were included in the model as a categorical variable. Other explanatory variables tested included; feed-genetic group (low-forage control: LF-C, low-forage select: LF-S, high forage control: HF-C, high forage select: HF-S), maximum age at first calving (< 24 months, 24 to 27 months, 28 to 30 months, 31 to 33 months and greater than 33 months).

Model fit was evaluated using conventional plots of standardised residuals and by examining the influence and leverage of data points (Rabash et al., 2009).

#### 2.2.3 Model 3: impacts of lesions present around first calving on culling

A discrete time survival model was used to explore the relationship between lesions and survival to culling. The model took the form;

Cull_ij_ ∼ Bernoulli (probability = π_ij_)

Logit(π_ij_) = α + β_1_wk_ij_ + β_2_X_ij_ + β_3_X_j_ + u_j_

[u_j_] ∼ N(0, σ^2^_v_)

Where subscripts *i* and *j* denote the _i_th observation of the _j_th cow respectively. π_ij_ = probability of a lame outcome for the _i_th observation of the _j_th cow. α = intercept value, wk_ij_ = categorical variable to represent week of the study for the _i_th observation of the _j_th cow, β_1_ = vector of coefficients for wk_ij_, X_ij_ = vector of covariates associated with each observation, β_2_ = coefficients for covariates X_ij,_ X_j_ = vector of covariates associated with each cow, β_3_ = coefficients for covariates X_j_. u_j_ = random effect to account for residual variation between cows (assumed to be normally distributed with mean = 0 and variance = σ^2^_v_).

Lesion scores were included as a categorical explanatory variable. Other potentially confounding explanatory variables tested were the same as described above for model 1. The only difference being that parity was not included and WIM was categorised in two 16-week intervals from 0 to 32 weeks and another category for > 32 weeks.

Posterior predictions were used to assess model fit by visual comparison to the observed data (Gelman et al., 1996) and the Hosmer-Lemeshow test (Hosmer and Lemeshow, 1989) was used as a statistical test for goodness of fit for logistic regression models.

## 3 Results

Data were available for a total of 158 heifers calving for the first time between August 2003 and March 2006; parity number ranged from 1 to 7 for animals in the complete dataset.

### 3.1 Descriptive analysis

**Fig. 1:**
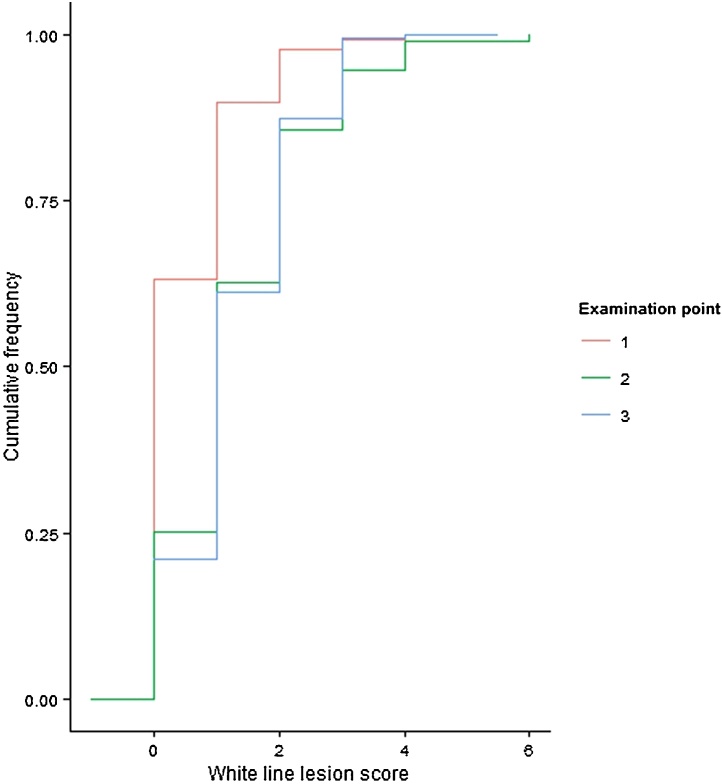
Cumulative frequency plot for white line lesion scores within each examination point for 158 heifers calving during the time period August 2003 to March 2006 and lesion scored during the period September 2003 to January 2006 at the Scotland’s Rural College (SRUC) Dairy Research and Innovation Centre. Examination points 1 to 3 represent; 1 = 0 to 2 months pre-calving, 2 = 0 to 2 months post-calving and 3 = 2 to 4 months post-calving.

**Fig. 2:**
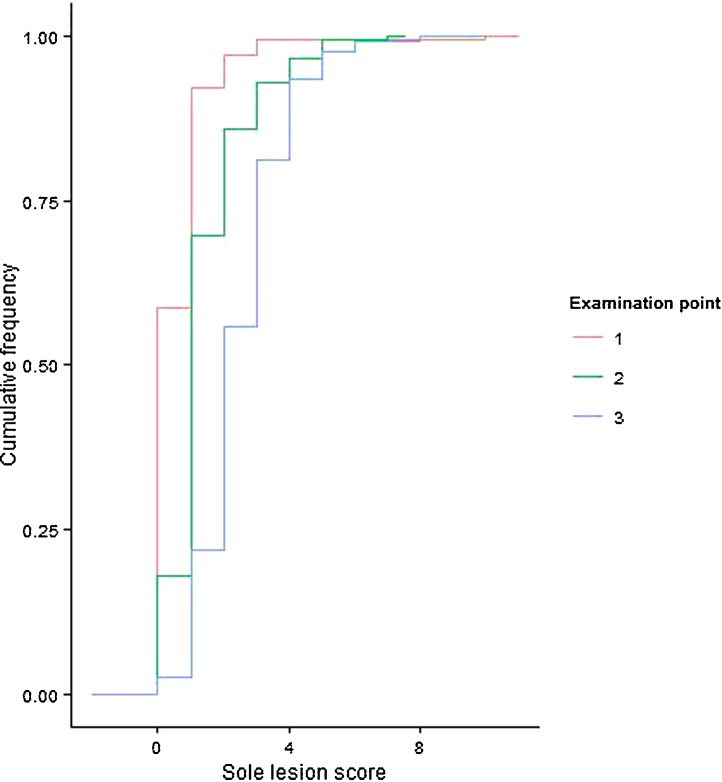
Cumulative frequency plot for sole lesion scores within each examination point for 158 heifers calving during the time period August 2003 to March 2006 and lesion scored during the period September 2003 to January 2006 at the Scotland’s Rural College (SRUC) Dairy Research and Innovation Centre. Examination points 1 to 3 represent; 1 = 0 to 2 months pre-calving, 2 = 0 to 2 months post-calving and 3 = 2 to 4 months post-calving.

Sole lesions were the most commonly observed lesion at each of the examination points and the proportion of heifers with sole lesions increased from pre-calving to 2 to 4 months post-calving, such that by 2 to 4 months post-calving 97% of heifers had some degree of sole lesion recorded (Table 3). A similar pattern of increasing proportions of heifers having a lesion recorded for each of the time periods was also observed for white line lesions; at the 2 to 4 month post-calving observation 81% of heifers had a lesion recorded (Table 3). Score severity also increased over the time periods 0 to 2 months pre-calving through to 2 to 4 months post-calving for white line and sole lesions (Fig. 1, Fig. 2).

Sole lesion and white line lesion scores were moderately correlated at each examination point (Kendall’s tau = 0.24, 0.35 and 0.13 for the time period 0 to 2 months pre-calving, 0 to 2 months post-calving and 2 to 4 months post-calving respectively, P ≤ 0.05).

### 3.2 Modelling

For all models, maximum lesion scores were included as a categorical variable in the final models. This was because the maximum score was considered biologically most likely to have an impact on subsequent health. Results for each of the models are described in detail below;

#### 3.2.1 Model 1: impacts of lesions present around first calving on lameness

The dataset included a total of 24,335 cow weeks at risk for 158 heifers with lesion score data. There were 4,093 lame events recorded in a total of 146 animals over the period September, 1, 2003 to August, 31, 2011. Table 4 shows the results from Model 1.

Heifers with white line lesion scores between 2 and 4 in the 0 to 2 months pre-calving period had a decreased risk of future lameness events compared with heifers with lesion score zero or 1 at this examination point (OR (95% credible interval) = 0.34 (0.13 to 0.86) for lesion score = 2 to 4).White line lesions with a score of ≥ 3 in the 2 to 4 months post-calving period were associated with a significantly increased risk of future lameness compared with a baseline of score of zero to 1 (OR (95% credible interval) = 3.48 (1.34 to 9.07) for score 3 to 4). Compared with the baseline white line lesion score of zero to 1, a score ≥ 3 at this examination point had a predicted increased relative risk of future lameness of 1.6.

More severe sole lesions (score of 4 to 8) in the 2 to 4 months post-calving were similarly associated with an increased risk of future lameness compared with a baseline score of 2 (OR (95% credible interval) = 2.90 (1.54 to 5.46) for scores ≥ 4). Heifers with lesion score zero or 1 in the 2 to 4 months post-calving were also at increased risk of future lameness compared with those with a mild lesion of score 2 (OR (95% credible interval) = 2.28 (1.16 to 4.48)). Compared with a baseline sole lesion score of 2, more severe sole lesions (score 4 to 8) at this time point had a predicted increased relative risk of future lameness of 2.6, whilst a score of zero or 1 had a predicted increased relative risk of future lameness of 2.1. Interactions between feed-genetic group and white line lesion score 2 to 4 months post-calving were significant including, Dry:C and score 2 (OR (95% credible interval) = 2.05 (1.08 to 3.90), HF:S and score 3 to 4 (OR (95% credible interval) = 0.21 (0.05 to 0.88) and Other:C and score 3 to 4 (OR (95% credible interval) = 0.06 (0.01 to 0.50). The variance at cow level was 0.85; inclusion of random effects improves model fit. Model fit was good, χ^2^ = 11.95, p = 0.22.

#### 3.2.2 Model 2: milk yield over lifetime within the Langhill herd

Milk yield data were available for 157 heifers, with an average time within the Langhill herd of 3.9 years. The mean (SD) average daily milk yield was 27.6 (5.9) kg with a range of 4.1 kg to 41.1 kg, and was approximately normally distributed. Table 5 shows the results for Model 2. The mean effect and mean number of days cows spent in the herd for each lesion category were used to calculate an adjusted yield loss for lesion categories.

**Fig. 3:**
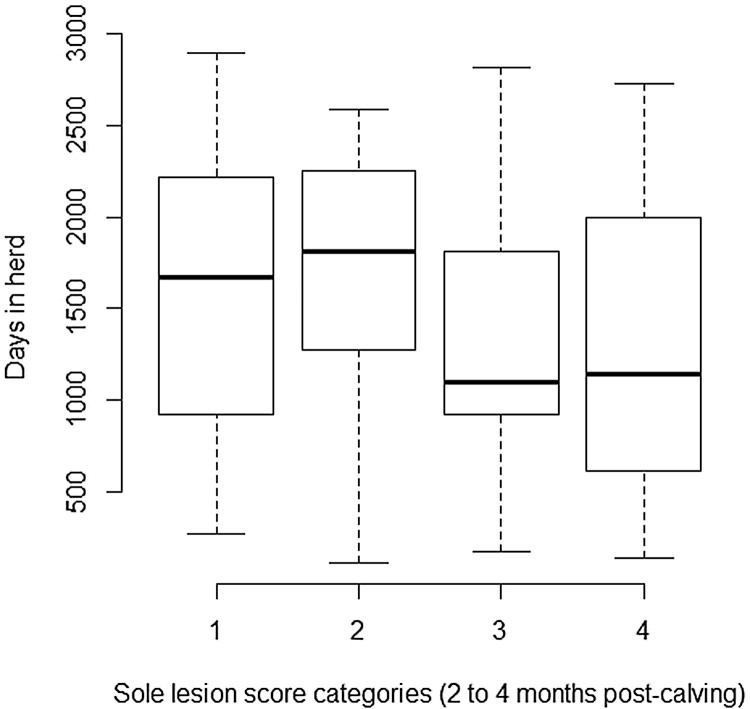
Box plot showing median and interquartile ranges for number of days in herd for each sole lesion category 2 to 4 months post-calving (lesion scores for categories; 1 = 0 to 1, 2 = 2, 3 = 3, 4 = 4 to 8) for 157 heifers calving during the time period August 2003 to March 2006 and lesion scored during the period September 2003 to January 2006 at the Scotland’s Rural College (SRUC) Dairy Research and Innovation Centre.

**Fig. 4:**
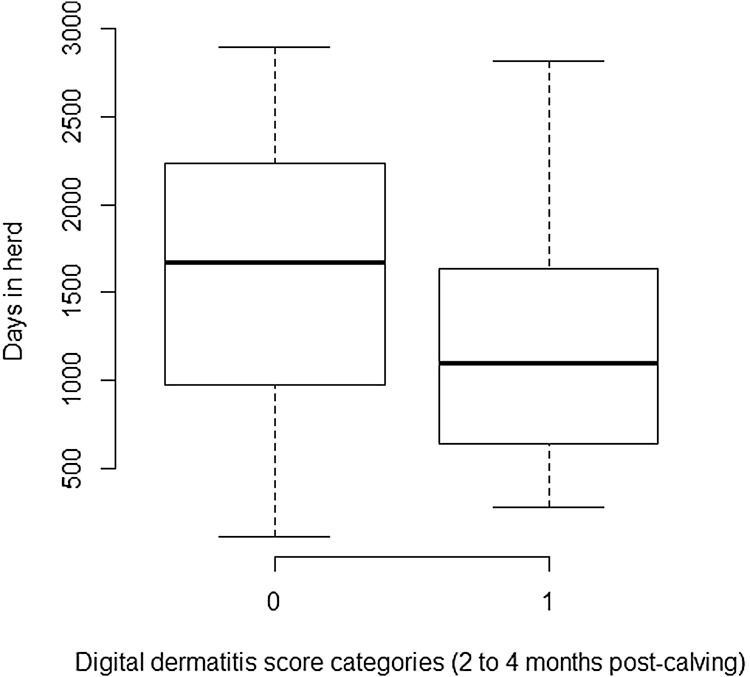
Box plot showing median and interquartile ranges for number of days in herd for the presence or absence of digital dermatitis 2 to 4 months post-calving (lesion scores for categories; 0 = lesion absent, 1 = lesion present) for 157 heifers calving during the time period August 2003 to March 2006 and lesion scored during the period September 2003 to January 2006 at the Scotland’s Rural College (SRUC) Dairy Research and Innovation Centre.

Heifers with sole lesions score ≥4 in the 2 to 4 month post-calving period had a significantly reduced average daily milk yield of 2.68 kg compared with those with no lesion at this time point. Animals with sole lesions score≥4 at this examination point remained within the herd a median of 326 days less (Fig. 3), therefore the mean yield loss associated with these sole lesions equated to 9,928 kg over the animals’ productive lifespan within the herd (calculated from the coefficient of the intercept multiplied by the mean number of days in the herd for cows in the baseline category minus the mean effect of the significant category multiplied by the mean number of days in the herd for cows in the significant category i.e (1631.88 × 19.72) − (1305.89 x (19.72+-2.68)). Digital dermatitis in the 2 to 4 months post-calving was associated with an increased average daily milk yield of 2.63 kg compared with no lesion. However, since animals with digital dermatitis lesions at this examination point remained in the herd for an average of 341 days less (Fig. 4) than those with no lesion at this examination point, the adjusted yield difference associated with the presence of digital dermatitis lesions compared with no lesion was a net loss of 3,513 kg of lifetime production within the herd (calculated using the method described above). Model fit was good.

#### 3.2.3 Model 3: impacts of lesions present around first calving on culling

Culling data were available for 157 heifers; 139 animals were culled within the study period September, 1, 2003 to August, 31, 2011. The data set included a total of 39,417 cow weeks at risk. Table 6 shows the results for Model 3.

**Fig. 5:**
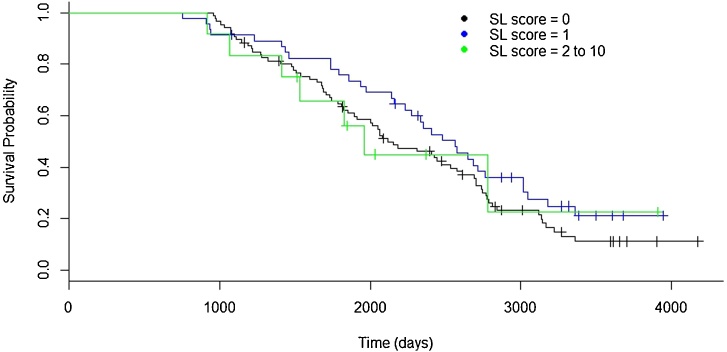
Kaplan-Meier survival plot for sole lesion (SL) categories 0 to 2 months pre-calving for 157 heifers calving during the time period August 2003 to March 2006 and lesion scored during the period September 2003 to January 2006 at the Scotland’s Rural College (SRUC) Dairy Research and Innovation Centre.

Sole lesions in the 0 to 2 months pre-calving was the only lesion in any of the time periods investigated with a significant association with culling (Fig. 5). Sole lesion of score 1 compared with no lesion was associated with a reduced risk of culling (OR (95% CI) = 0.52 (0.32 to 0.84)). The variance at cow level was 0.61; inclusion of random effects improves model fit. Model fit was good, χ^2^ = 0.55, p = 0.76.

## 4 Discussion

This study reports on the long term impacts of foot lesions around the time of first calving in heifers, on future lameness risk, milk yield and culling risk in a dairy herd. Previous studies investigating the impacts of lameness in heifers on these outcomes have looked separately at impacts on future lameness (Hirst et al., 2002) or milk yield (Onyiro et al., 2008), or at impacts of digital dermatitis in pre-calving heifers on culling restricted to the first lactation (Gomez et al., 2015). As heifers represent the future of the dairy herd, understanding the overall effects of lesions occurring around the time of first calving could be important for improving lameness control in dairy herds. A particularly novel finding from this study was the reduced risk of culling associated with mild sole lesions (score 1) in the 0 to 2 months pre-calving.

### 4.1 Impacts of lesions present around first calving on culling

To the authors’ knowledge, there are no previous studies reporting the impacts of lesions in heifers on long term survival within the dairy herd. Gomez et al. (2015) reported a numerically, but not statistically significant effect of digital dermatitis in pre-calving heifers on increased risk of culling before 60 days in milk (DIM) in their first lactation. Sogstad et al. (2007) used claw trimming data from 500 Norwegian herds to investigate the impacts of lameness and lesions on culling within the same lactation that claw trimming took place; lameness in lactation 1 was associated with earlier culling (hazard ratio = 4.2). Previous studies have reported significant negative effects of lameness in adult dairy cows on survival (Booth et al., 2004, Bicalho et al., 2007, Machado et al., 2010). Whilst Barkema et al. (1994) reported a lower culling rate associated with lameness, thought to be attributed to the retention of lame cows because of the higher milk production of these cows. Interpreting survival within the herd is complex due to the decisions behind culling, which may be a direct response to lameness or due to indirect effects of lameness on milk yield and fertility, alongside many other health and management reasons. In the current study the data were analysed using time to culling for all reasons due to this uncertainty. The findings of the current study suggest that mild sole lesions occurring at a time when the animal is able to recover from and adapt to the insult may be beneficial for long term survival. Since the reasons for culling were not analysed it is not possible to identify possible underlying mechanisms, however this finding is consistent with some of the other outcomes explored; mild sole lesions and white line lesions were also associated with a reduced risk of lameness. Therefore further research is required to understand the underlying mechanisms associated with this finding and to clarify the extent to which mild lesions may offer protection.

### 4.2 Impacts of lesions present around first calving on future lameness

In the current study, more severe white line lesions and sole lesions were associated with a significantly increased risk of future lameness by 1.6 and 2.6 times respectively, across all future lactations within the herd. These results are similar to Hirst et al. (2002) who reported a positive association between claw horn lameness in heifers and future risk, but only for the second lactation; this association was not significant for the third lactation. Hirst et al. (2002) also found that any type of lameness in the first lactation was associated with claw-horn lameness in the second lactation and hypothesised that this may be due to underlying pathology that is carried over from one lactation to the next. This hypothesis is supported by the findings of a study which used micro-computed tomography and reported that claw horn lesions during life were associated with an increase in pathological changes to the bony architecture of the pedal bone (Newsome et al., 2016). This could explain the relationship between more severe claw horn lesions and future lameness risk observed in this study. Further work is required to understand the longitudinal relationship between causal factors and the role of pathological changes to distal limb anatomy associated with claw horn lesions.

In the current study, mild sole lesion and white line lesions occurring in the 2 to 4 months post-calving or 0 to 2 month pre-calving periods respectively, were associated with a reduced risk of lameness. This suggests that some degree of mild insult around the time of first calving may be beneficial to long term claw health; if adaptive changes occur in response to the insult during a time when the claw is able to recover and become more biomechanically resilient the animal may be less prone to lameness in the long term. Bergsten et al. (2015) reported findings that support this hypothesis; heifers reared on a hard flooring surface (cubicles with slatted concrete alleys) pre-calving and housed on a soft surface (slatted rubber alleys) post-calving resulted in the lowest prevalence and severity of sole and white-line haemorrhages in first-lactation. The authors suggested that the challenge from hard flooring during the rearing period resulted in traumatic sole haemorrhages, but as the heifers were able to cope at this time, this was ultimately beneficial for claw health. Previous studies have demonstrated that adaptive changes can take place in the bovine hoof and indicate that environment and exercise have a role in the development of the hoof support structures (Knott et al., 2007, Gard et al., 2015). Additional research is required to understand the mechanisms underlying these findings and therefore their clinical relevance. The findings of this study suggest that there may be a threshold for severity of white line and sole lesions that is associated with an increased risk of future lameness, but that some degree of mild insult occurring at certain times may initiate adaptive changes within the hoof that are beneficial to long term claw health. Husbandry practices implemented during the pre- and post-calving period may have significant impacts on future lameness in adult cattle as a result of reducing the more severe lesions occurring during this time. Additionally, management of husbandry practices may also allow for adaptive changes to occur in the hoof that best prepare those heifers for their future life in the herd.

The interaction between the feed-genetic group HF:S and white line disease 2 to 4 months post-calving suggests that there may be environmental or nutritional factors mitigating the impacts of sole lesions occurring during this time period on the risk of future lameness. It may therefore be possible that the effects of claw horn lesions are different in herds with different management systems, for example grazed vs continuously housed.

The high prevalence of lesions in heifers has previously been highlighted in a number of studies (Manske et al., 2002, Capion et al., 2009, Maxwell et al., 2015), and at a similar level to that observed in this study. This is relevant not only for the health and welfare of those animals affected at that time, but also when considering the impacts on future health and welfare.

### 4.3 Impacts of lesions present around first calving on milk yield

Severe sole lesions 2 to 4 months post-calving were associated with a reduction in average daily yield of 2.68 kg in the current study. A number of studies have demonstrated a reduction in milk yield associated with lameness in dairy cows of all ages (Green et al., 2002, Amory et al., 2008, Archer et al., 2010). Amory et al. (2008) investigated the effect of lesion-specific causes of lameness on milk yield in 1824 UK dairy cows. Sole ulcer and white line disease were associated with a lactation milk yield loss of approximately 570 and 370 kg respectively, whilst a slight increase in yield was observed following treatment of digital dermatitis. Interpretation of milk yield losses associated with lameness can be difficult, as it is the higher yielding cows that are more likely to become lame (Green et al., 2002, Amory et al., 2008, Archer et al., 2010). This association may also lead to retention of lame cows within the herd. Maxwell et al. (2015) reported that lame heifers produced significantly more milk over the first lactation (734 l, P = 0.02) than those that were not lame. In a study carried out 2003 to 2005 on the same UK research herd as the current study, no association was found between lameness in heifers and their 305-day yield. The study did not however explore the longitudinal relationship between lameness in heifers and their long-term future milk yield (Onyiro et al., 2008). The results of the current study demonstrate that severe sole lesions were associated with a long-term negative impact on milk yield. This population of animals also remained within the herd on average nearly one year less than those with no lesion. Similarly, as animals with DD lesions remained within the herd for a shorter period, DD was associated with a net yield loss, despite positive associations between DD and yield in this study and as reported by Amory et al. (2008). Gomez et al. (2015) also demonstrated that animals with DD lesions during the rearing period produce significantly less milk during their subsequent lactation. The reasons for cows leaving the Langhill herd were not recorded, therefore it is not possible to explain why severe sole lesions and DD were associated with a shorter time period within the Langhill herd.

### 4.4 Study limitations and generalisability

One of the limitations associated with this study in demonstrating the impacts of early life lesions in heifers on future lameness and performance, was that the regular examination of claws may have increased the level of treatment interventions, compared with the situation more commonly observed on UK commercial dairy farms. This may have resulted in a reduced effect of lesions on future lameness, milk yield and culling in this herd.

The published literature includes supporting evidence for the reduction in milk yield and longevity reported here and the possible protective effects of mild foot lesions on future robustness. We conclude that whilst this study was carried out on one UK dairy herd and the quantitative impact of severe foot lesions in heifers are likely to be specific to this study, the qualitative impacts are likely to be generic across dairy herds using similar management systems.

Whilst the lesion scoring systems are subjective and may be prone to inter-observer variability, lesion scoring within the study period was limited to one person and any variability should not undermine the conclusions of the study findings. The authors also acknowledge the lack of a widely accepted lesion scoring system and the issues associated with a lack of understanding of pathogenesis and therefore lesion progression associated with claw horn lesions. Consequently results should be interpreted with consideration of the approach taken. Finally, due to the difficulties in defining the duration of a case of lameness (especially in situations where lameness scoring is conducted frequently), in this paper the risk of a cow being lame in any one week was modelled and results should therefore be interpreted in this context.

## 5 Conclusions

This study demonstrated that mild sole lesions are associated with an overall reduced risk of premature culling in dairy cows. We hypothesise that a mild insult may be beneficial to claw health; if adaptive changes occur in response to the insult during a time when the claw is able to recover and become more biomechanically resilient. High and low scores for white line and sole lesions in heifers were associated with a greater risk of future lameness than medium scores. High sole lesion scores and digital dermatitis were associated with an overall reduction in milk yield when adjusted for mean time within the herd. We conclude that the current high prevalence of more severe claw horn lesions in dairy heifers is likely to have a large impact on the health, welfare and productivity of these animals over their lifetime within the herd. Identifying and implementing husbandry practices which reduce the occurrence of severe claw horn lesions is essential for the future sustainability of dairy herd production.

## Conflicts of interest

none

## Figures and Tables

**Table 1:** Example young-stock ration fed to dairy heifers calving during the time period August 2003 to March 2006 at the Scotland’s Rural College (SRUC) Dairy Research and Innovation Centre.

Description	DM (%)	Actual Weight (kg/animal)	
Young-stock ration	54.66	16.10	
		Actual	
		kg/animal	% Load
Straw	80.00	6.00	37.27
Distillery co-product	30.00	8.00	49.69
General purpose minerals	100.00	0.10	0.62
Molasses	75.00	2.00	12.42

**Table 2:** Description of severity scores for hoof lesions recorded for heifers calving during the time period August 2003 to March 2006 and lesion scores recorded during the period September 2003 to January 2006 in the SRUC Dairy Research and Innovation Centre 2003 to 2006 (Offer et al., 2000, Leach et al., 2005).

Visual appearance of lesion	Lesion score	Number of heifers with maximum lesion score recorded in each examination period		
		0–2 months pre-calving	0–2 mths post-calving	2–4 months post-calving
Sole lesion				
Diffuse red or yellow in horn	1	40	57	21
Defined red in hoof horn	2	8	20	32
Stronger red colouration	3	4	8	31
Deep dense red	4	0	5	17
Port coloured	5	0	5	7
Mild sole ulcer, possible fresh blood	6	0	0	2
Corium exposed	7	0	2	0
Corium exposed with some loss of horn	8	0	0	1
Deep sole ulcer with major horn loss	9	0	0	0
Infected sole ulcer	10	1	0	0

White line lesion				
Diffuse red or yellow in white line	1	32	45	44
Defined red in white line	2	12	26	32
Stronger red colouration	3	2	13	16
Deep dense red	4	1	5	1
Port coloured	5	0	0	0
Separation of the white line, possible fresh blood	6	0	1	0
Corium exposed with separation	7	0	0	0
Corium exposed and loss of horn with separation	8	0	0	0
Deep separation of the white line	9	0	0	0
Infection present in the white line	10	0	0	0

Digital Dermatitis				
Lesion present in a small area	1	5	9	5
Larger lesion with slight exudate	2	4	4	11
Deeper lesion with exudate reddening and swelling	3	4	5	4

**Table 3:** Summary of the number of hoof lesions recorded for each examination point and lesion cumulative incidence in 158 heifers calving during the time period August 2003 to March 2006 and lesion scored during the period September 2003 to January 2006 at the Scotland’s Rural College (SRUC) Dairy Research and Innovation Centre.

Lesion	Examination point	Total number of heifers observed^a^	Number of heifers with lesions on at least one claw	Lesion cumulative incidence
White line lesion	0 to 2 months pre-calving	145	57	0.39
	0 to 2 months post-calving	128	96	0.75
	2 to 4 months post-calving	118	95	0.81
Sole lesion	0 to 2 months pre-calving	145	59	0.41
	0 to 2 months post-calving	128	103	0.80
	2 to 4 months post-calving	118	115	0.97
Digital dermatitis	0 to 2 months pre-calving	145	14	0.10
	0 to 2 months post-calving	128	19	0.15
	2 to 4 months post-calving	118	20	0.17

a A total of 158 heifers were included in the data set, however actual examination periods and categorised examination periods are likely to not always coincide, therefore resulting in missing observations. We have no other information to suggest these data were anything other than missing at random.

**Table 4:** Model 1: Binomial model for repeated lameness events in 158 heifers calving during the time period August 2003 to March 2006 and lesion scored during the period September 2003 to January 2006, with herd lifetime data recorded from September 2003 to August 2011 in the SRUC Dairy Research and Innovation Centre herd.

Intercept			Coefficient: − 4.49		
Variable	N^1^	Lame^2^	Odds ratio	Lower 95% CrI^3^	Upper 95% CrI

White line lesion score (0 to 2 months pre-calving)					
0 to 1^4a^	20,058	0.17	Baseline		
2 to 4^4b^	2,272	0.13	0.34	0.13	0.86

White line lesion score (2 to 4 months post-calving)					
0 to 1^4c^	11,781	0.15	Baseline		
2^4d^	5,035	0.16	1.48	0.70	3.12
3 to 4^4e^	2,434	0.19	3.48	1.34	9.07

Sole lesion (2 to 4 months post-calving)					
2^4f^	6,522	0.14	Baseline		
0 to 1^4g^	4,226	0.18	2.28	1.16	4.48
3^4h^	4,897	0.14	1.53	0.87	2.67
4 to 8^4i^	3,605	0.20	2.90	1.54	5.46

Feed − genetic group^5^					
LF:C	4,758	0.15	Baseline		
LF:S	4,757	0.21	1.04	0.50	2.16
HF:C	5,462	0.13	0.99	0.62	1.58
HF:S	5,680	0.17	1.52	0.73	3.13
Dry:C	1,436	0.14	0.90	0.53	1.52
Dry:S	1,273	0.21	2.33	1.03	5.27
Other:C	137	0.55	7.62	3.17	18.30
Other:S	183	0.62	12.82	4.79	34.29

Locomotion score assessor (1 to 4)					
1	1,150	0.42	Baseline		
2	11,331	0.11	0.38	0.30	0.48
3	11,116	0.17	1.12	0.88	1.41
4	738	0.69	1.93	1.34	2.79

Week category^6^					
0–30	1,285	0.09	Baseline		
31–60	1,889	0.06	0.72	0.50	1.02
61–90	2,595	0.07	0.64	0.46	0.89
91–120	3,031	0.08	0.66	0.48	0.92
121–150	3,596	0.11	1.05	0.76	1.46
151–180	3,351	0.13	1.36	0.98	1.89
181–210	2,864	0.13	1.71	1.21	2.43
211–240	2,367	0.25	4.27	3.03	6.00
241–300	2,507	0.43	6.10	4.26	8.74
301–360	733	0.59	8.53	5.24	13.90
>360	117	0.69	6.21	2.85	13.52

Weeks in milk					
0–8	3,765	0.14	Baseline		
9–16	3,457	0.15	1.16	0.95	1.42
17–24	2,963	0.17	1.53	1.24	1.89
25–32	2,496	0.18	1.56	1.25	1.95
32–40	2,876	0.20	1.59	1.27	1.97
>40	5,117	0.14	1.33	1.10	1.61

Previous lameness event (0 to 2 months)^7^					
None	10,230	0.03	Baseline		
Lameness event	12,628	0.28	3.96	3.37	4.65

Previous lameness event (2 to 4 months)					
None	9,737	0.06	Baseline		
Lameness event	11,854	0.27	1.51	1.31	1.73

Number lame weeks per cow^8^					
≤ 40 lameness events	17,240	0.09	Baseline		
>40 lameness events	7,095	0.36	3.70	2.30	5.97

Feed − genetic group x White line lesion score (2 to 4 months post-calving)					
LF:S x 2			1.12	0.37	3.40
HF:C x 2			1.00	0.47	2.11
HF:S x 2			1.00	0.34	2.94
Dry:C x 2			2.05	1.08	3.90
Dry:S x 2			2.23	0.74	6.75
Other:C x 2			0.39	0.12	1.27
Other:S x 2			1.63	0.33	8.06
LF:S x 3 to 4			0.58	0.15	2.27
HF:C x 3 to 4			0.38	0.11	1.33
HF:S x 3 to 4			0.21	0.05	0.88
Dry:C x 3 to 4			0.99	0.33	2.97
Dry:S x 3 to 4			0.35	0.08	1.46
Other:C x 3 to 4			0.06	0.01	0.50
Other:S x 3 to 4			0.58	0.03	12.60
Random effect			Variance: 0.85		

^1^N = Total number of observations (cow weeks) within each category.

^2^Proportion of observations recorded lame within each category.

^3^CrI = credible interval.

^4a toi^Number of cows with lesions observed within each lesion score category; a = 118, b = 15, c = 65, d = 32, e = 17, f = 32, g = 2, h = 31, i = 27.

^5^Feed-genetic groups include low forage (LF), high forage (HF), control (C), and select (S). Dry refers to dry cows, and other refers to all other management groups outside of LF, HF, and Dry.

^6^Week category = week of the study period, included as a categorical variable.

^7^Previous lameness event based on locomotion score recorded as 3, 4 or 5.

^8^Covariate for number of lame weeks per cow (>40) was added to the model to correct model over-dispersion and improve model fit.

**Table 5:** Model 2: Linear regression model for average daily milk yield within the Langhill herd in 157 heifers calving during the time period August 2003 to March 2006, with herd lifetime data recorded from September 2003 to August 2011 at the SRUC Dairy Research and Innovation Centre herd.

Intercept		Coefficient: 19.72		
Variable	N^a^	Mean effect	Lower 95% CI^b^	Upper 95% CI
Sole Lesion (2 to 4 months post-calving)				
0 to 1	24	Baseline		
2	35	−0.76	−3.03	1.50
3	32	0.008	−2.29	2.30
4 to 8	27	−2.68	−5.05	−0.31
Digital dermatitis (2 to 4 months post-calving)				
0	98	Baseline		
1 to 3	20	2.63	0.51	4.75
Feed − genetic group^c^				
LF:C	30	Baseline		
LF:S	36	10.57	7.99	13.14
HF:C	30	−1.41	−3.87	1.05
HF:S	32	2.737	0.27	5.21

a N = Number of heifers with lesions observed within each category.

b CI = confidence interval. Parameter is ‘significant' if the 95% confidence interval excludes 0.

c Feed-genetic groups include low forage (LF), high forage (HF), control (C), and select (S).

**Table 6:** Model 3: Binomial model for survival to culling in 157 heifers calving during the time period August 2003 to March 2006, with herd lifetime data recorded from September 2003 to August 2011 at the SRUC Dairy Research and Innovation Centre.

Intercept		Coefficient: 9.18		
Variable	N^1^	Odds ratio	Lower 95% CrI^2^	Upper 95% CrI
Sole lesion (0 to 2 months pre-calving)				
0^3a^	17,096	Baseline		
1^3b^	10,709	0.52	0.32	0.84
2 to 10^3c^	2,164	0.70	0.31	1.61

Feed − genetic group^4^				
LF:C	4,728	Baseline		
LF:S	4,927	2.27	0.93	5.50
HF:C	5,625	0.45	0.14	1.44
HF:S	5,833	0.76	0.24	2.39
Dry:C	1,801	0.37	0.13	1.08
Dry:S	1,593	0.66	0.23	1.86
Other:C	5,187	1.39	0.51	3.80
Other:S	1,924	3.97	1.39	11.39

Week category^4^				
0–60	3,362	Baseline		
61–120	5,923	1.08	0.31	3.72
121–180	7,552	2.09	0.62	7.11
181–240	5,825	2.11	0.57	7.81
241–300	4,604	1.04	0.24	4.42
301–360	3,262	1.48	0.32	6.77
>360	1,889	1.00	0.18	5.46

Weeks in milk				
0–16	8,357	Baseline		
17–32	6,239	1.89	0.69	5.16
>32	9,211	2.58	1.06	6.30
Random effect		Variance: 0.61		

^1^N = Number of cow weeks.

^2^CrI = credible interval.

^3a to c^Number of cows with lesions observed within each lesion score category; a = 78, b = 38, c = 11.

^4^Feed-genetic groups include low forage (LF), high forage (HF), control (C), and select (S). Dry refers to dry cows, and Other refers to all other management groups outside of LF, HF, and Dry.

^5^Week category = week of the study period, included as a categorical variable.

## References

[bib0005] Amory J.R., Barker Z.E., Wright J.L., Mason S.A., Blowey R.W., Green L.E. (2008). Associations between sole ulcer, white line disease and digital dermatitis and the milk yield of 1824 dairy cows on 30 dairy cow farms in England and Wales from February 2003-November 2004. Prev. Vet. Med..

[bib0010] Archer S.C., Green M.J., Huxley J.N. (2010). Association between milk yield and serial locomotion score assessments in UK dairy cows. J. Dairy. Sci..

[bib0015] Archer S.C., Mc Coy F., Wapenaar W., Green M.J. (2013). Association between somatic cell count after first parturition and cumulative milk yield in dairy cows. Vet. Rec..

[bib0020] Barkema H.W., Westrik J.D., Keulen K.A.S.v., Schukken Y.H., Brand A. (1994). The effects of lameness on reproductive performance, milk production and culling in Dutch dairy farms. Prev. Vet. Med..

[bib0025] Bell N.J., Bell M.J., Knowles T.G., Whay H.R., Main D.J., Webster A.J.F. (2009). The development, implementation and testing of a lameness control programme based on HACCP principles and designed for heifers on dairy farms. Vet. J..

[bib0030] Bergsten C., Telezhenko E., Ventorp M. (2015). Influence of soft or hard floors before and after first calving on dairy Heifer locomotion, claw and leg health. Animals.

[bib0035] Bicalho R.C., Vokey F., Erb H.N., Guard C.L. (2007). Visual locomotion scoring in the first seventy days in milk: impact on pregnancy and survival. J. Dairy Sci..

[bib0040] Booth C.J., Warnick L.D., Grohn Y.T., Maizon D.O., Guard C.L., Janssen D. (2004). Effect of lameness on culling in dairy cows. J. Dairy Sci..

[bib0045] Capion N., Thamsborg S.M., Enevoldsen C. (2009). Prevalence and severity of foot lesions in Danish Holstein heifers through first lactation. Vet. J..

[bib0050] Chagunda M.G.G., Römer D.A.M., Roberts D.J. (2009). Effect of genotype and feeding regime on enteric methane: non-milk nitrogen and performance of dairy cows during the winter feeding period. Livest. Sci..

[bib0055] Defra, (2003). Code of Recommendations for the Welfare of Livestock.

[bib0060] Dohoo I., Martin W., Stryhn H. (2003). Veterinary Epidemiologic Research.

[bib0065] Gard J.A., Taylor D.R., Wilhite D.R., Rodning S.P., Schnuelle M.L., Beyers R.J., Edmonson M.A., DeGraves F.J., van Santen E. (2015). Effect of exercise and environmental terrain on development of the digital cushion and bony structures of the bovine foot. Am. J. Vet. Res..

[bib0070] Gelman A., Meng X., Stern H. (1996). Posterior predictive assessment of model fitness via realized discrepancies. Statistica Sin..

[bib0075] Goldstein, H., 1995. Multilevel Statistical Models. London: Edward Arnold New York: Halstead Press.

[bib0080] Goldstein H. (2003). Multilevel Statistics Models.

[bib0085] Gomez A., Cook N.B., Socha M.T., Dopfer D. (2015). First-lactation performance in cows affected by digital dermatitis during the rearing period. J. Dairy. Sci..

[bib0090] Green L.E., Hedges V.J., Schukken Y.H., Blowey R.W., Packington A.J. (2002). The impact of clinical lameness on the milk yield of dairy cows. J. Dairy. Sci..

[bib0095] Green M.J., Burton P.R., Green L.E., Schukken Y.H., Bradley A.J., Peeler E.J., Medley G.F. (2004). The use of Markov chain Monte Carlo for analysis of correlated binary data: patterns of somatic cells in milk and the risk of clinical mastitis in dairy cows. Prev. Vet. Med..

[bib0100] Green L.E., Huxley J.N., Banks C., Green M.J. (2014). Temporal associations between low body condition, lameness and milk yield in a UK dairy herd. Prev. Vet. Med..

[bib0105] Greenough P.R., Vermunt J.J. (1991). Evaluation of subclinical laminitis in a dairy herd and observations on associated nutritional and management factors. Vet. Rec..

[bib0110] Hirst W.M., Murray R.D., Ward W.R., French N.P. (2002). A mixed-effects time-to-event analysis of the relationship between first-lactation lameness and subsequent lameness in dairy cows in the UK. Prev. Vet. Med..

[bib0115] Hosmer D.W., Lemeshow S. (1989). Applied Logistic Regression.

[bib0120] Huxley J.N. (2013). Impact of lameness and claw lesions in cows on health and production. Livest. Sci..

[bib0125] Kendall M.G. (1955). Rank Correlation Methods.

[bib0130] Knott L., Tarlton J.F., Craft H., Webster A.J.F. (2007). Effects of housing, parturition and diet change on the biochemistry and biomechanics of the support structures of the hoof of dairy heifers. Vet. J..

[bib0135] Leach K.A., Offer J.E., Svoboda I., Logue D.N. (2005). Effects of type of forage fed to dairy heifers: associations between claw characteristics, clinical lameness, environment and behaviour. Vet. J..

[bib0140] Machado V.S., Caixeta L.S., McArt J.A.A., Bicalho R.C. (2010). The effect of claw horn disruption lesions and body condition score at dry-off on survivability, reproductive performance, and milk production in the subsequent lactation. J. Dairy. Sci..

[bib0145] Manske T., Hultgren J., Bergsten C. (2002). Prevalence and interrelationships of hoof lesions and lameness in Swedish dairy cows. Prev. Vet. Med..

[bib0150] Manson F.J., Leaver J.D. (1988). The influence of concentrate amount on locomotion and clinical lameness in dairy cattle. Anim. Prod..

[bib0155] Maxwell O.J., Hudson C.D., Huxley J.N. (2015). Effect of early lactation foot trimming in lame and non-lame dairy heifers: a randomised controlled trial. Vet. Rec..

[bib0160] Mulvany P.M. (1977). A body condition scoring technique for use with British Friesian cows. Anim. Prod..

[bib0165] Newsome R., Green M.J., Bell N.J., Chagunda M.G.G., Mason C.M., Sturrock C.J., Whay H.R., Huxley J.N. (2016). Linking bone development on the caudal aspect of the distal phalanx with lameness during life. J. Dairy. Sci..

[bib0170] Offer J.E., McNulty D., Logue D.N. (2000). Observations of lameness, hoof conformation and development of lesions in dairy cattle over four lactations. Vet. Rec..

[bib0175] Onyiro O.M., Offer J., Brotherstone S. (2008). Risk factors and milk yield losses associated with lameness in Holstein-Friesian dairy cattle. Animal.

[bib0180] Pryce J.E., Nielsen B.L., Veerkamp R.F., Simm G. (1999). Genotype and feeding system effects and interactions for health and fertility traits in dairy cattle. Livest. Prod. Sci..

[bib0185] Rabash J., Charlton C., Browne W.J., Healy M., Cameron B. (2009). MLwiN Version 2.1 Centre for Multilevel Modelling.

[bib0190] Randall L.V., Green M.J., Chagunda M.G.G., Mason C., Archer S.C., Green L.E., Huxley J.N. (2015). Low body condition predisposes cattle to lameness: an 8-year study of one dairy herd. J. Dairy Sci..

[bib0195] Rioja-Lang F.C., Roberts D.J., Healy S.D., Lawrence A.B., Haskell M.J. (2009). Dairy cows trade-off feed quality with proximity to a dominant individual in Y-maze choice tests. Appl. Anim. Behav. Sci..

[bib0200] Roberts D.J., March M. (2013). Feeding Systems for Dairy Cows: Homegrown Versus By-Product Feeds.

[bib0205] Sogstad Å.M., Østerås O., Fjeldaas T., Nafstad O. (2007). Bovine claw and limb disorders related to culling and carcass characteristics. Livest. Sci..

[bib0210] Tarlton J.F., Holah D.E., Evans K.M., Jones S., Pearson G.R., Webster A.J.F. (2002). Biomechanical and histopathological changes in the support structures of bovine hooves around the time of first calving. Vet. J..

[bib0215] Webster A.J. (2002). Effects of housing practices on the development of foot lesions in dairy heifers in early lactation. Vet. Rec..

