# Supplementary material for: Lameness in dairy heifers; impacts of hoof lesions present around first calving on future lameness, milk yield and culling risk
Source: Prev Vet Med. 2016 Oct 1;133:52–63. doi: 10.1016/j.prevetmed.2016.09.006 (PMC5063951; doi:10.1016/j.prevetmed.2016.09.006)
Supplement: Supplementary file 1 [file mmc1.docx]

SUPPLEMENTARY MATERIALS

Supplementary to Figure 1. Table of cumulative frequencies for white line lesion scores within each examination point for 158 heifers calving during the time period August 2003 to March 2006 and lesion scored during the period September 2003 to January 2006 at the Scotland’s Rural College (SRUC) Dairy Research and Innovation Centre. Examination points 1 to 3 represent; 1 = 0 to 2 months pre-calving, 2 = 0 to 2 months post-calving and 3 = 2 to 4 months post-calving.

| Examination point 1 |  |
| --- | --- |
| Lesion score | Cumulative frequency |
| 1 | 0.63 |
| 2 | 0.90 |
| 3 | 0.98 |
| 4 | 0.99 |
| 5 | 1.00 |
| 6 | 1.00 |
| 7 | 1.00 |
| 8 | 1.00 |
| 9 | 1.00 |
| 10 | 1.00 |
| Examination point 2 |  |
| Lesion score |  |
| 1 | 0.25 |
| 2 | 0.63 |
| 3 | 0.86 |
| 4 | 0.95 |
| 5 | 0.99 |
| 6 | 0.99 |
| 7 | 1.00 |
| 8 | 1.00 |
| 9 | 1.00 |
| 10 | 1.00 |
| Examination point 3 |  |
| Lesion score |  |
| 1 | 0.38 |
| 2 | 0.72 |
| 3 | 0.91 |
| 4 | 0.98 |
| 5 | 1.00 |
| 6 | 1.00 |
| 7 | 1.00 |
| 8 | 1.00 |
| 9 | 1.00 |
| 10 | 1.00 |

Supplementary to Figure 2. Table of cumulative frequencies for sole lesion scores within each examination point for 158 heifers calving during the time period August 2003 to March 2006 and lesion scored during the period September 2003 to January 2006 at the Scotland’s Rural College (SRUC) Dairy Research and Innovation Centre. Examination points 1 to 3 represent; 1 = 0 to 2 months pre-calving, 2 = 0 to 2 months post-calving and 3 = 2 to 4 months post-calving.

| Examination point 1 |  |
| --- | --- |
| Lesion score | Cumulative frequency |
| 1 | 0.59 |
| 2 | 0.92 |
| 3 | 0.97 |
| 4 | 1.00 |
| 5 | 1.00 |
| 6 | 1.00 |
| 7 | 1.00 |
| 8 | 1.00 |
| 9 | 1.00 |
| 10 | 1.00 |
| Examination point 2 |  |
| Lesion score |  |
| 1 | 0.18 |
| 2 | 0.70 |
| 3 | 0.86 |
| 4 | 0.93 |
| 5 | 0.96 |
| 6 | 1.00 |
| 7 | 1.00 |
| 8 | 1.00 |
| 9 | 1.00 |
| 10 | 1.00 |
| Examination point 3 |  |
| Lesion score |  |
| 1 | 0.38 |
| 2 | 0.72 |
| 3 | 0.91 |
| 4 | 0.98 |
| 5 | 1.00 |
| 6 | 1.00 |
| 7 | 1.00 |
| 8 | 1.00 |
| 9 | 1.00 |
| 10 | 1.00 |

Supplementary to Figure 3. Table showing mean number of days in herd for each sole lesion category identified 2 to 4 months post-calving (lesion scores for categories; 1 = 0 to 1, 2 = 2, 3 = 3, 4 = 4 to 8) for 157 heifers calving during the time period August 2003 to March 2006 and lesion scored during the period September 2003 to January 2006 at the Scotland’s Rural College (SRUC) Dairy Research and Innovation Centre.

| Lesion score category | Mean days in herd |
| --- | --- |
| 1 | 1632 |
| 2 | 1700 |
| 3 | 1344 |
| 4 | 1306 |

Supplementary to Figure 4. Table showing mean for number of days in herd for the presence or absence of digital dermatitis identified 2 to 4 months post-calving (lesion scores for categories; 0 = lesion absent, 1 = lesion present) for 157 heifers calving during the time period August 2003 to March 2006 and lesion scored during the period September 2003 to January 2006 at the Scotland’s Rural College (SRUC) Dairy Research and Innovation Centre.

| Lesion score category | Mean days in herd |
| --- | --- |
| 1 | 1558 |
| 2 | 1217 |
